# Gut Microbiota–Brain Axis as a Potential Modulator of Psychological Stress after Spinal Cord Injury

**DOI:** 10.3390/biomedicines10040847

**Published:** 2022-04-04

**Authors:** Samir Musleh-Vega, Jorge Ojeda, Pia M. Vidal

**Affiliations:** Neuroimmunology and Regeneration of the Central Nervous System Unit, Biomedical Science Research Laboratory, Basic Sciences Department, Faculty of Medicine, Universidad Católica de la Santísima Concepción, Concepción 4090541, Chile; smusleh@magister.ucsc.cl (S.M.-V.); jojeda@ucsc.cl (J.O.)

**Keywords:** spinal cord injury, psychological stress, microbiota–brain–gut axis

## Abstract

A growing body of evidence from preclinical and clinical studies has associated alterations of the gut microbiota–brain axis with the progression and development of a number of pathological conditions that also affect cognitive functions. Spinal cord injuries (SCIs) can be produced from traumatic and non-traumatic causes. It has been reported that SCIs are commonly associated with anxiety and depression-like symptoms, showing an incidence range between 11 and 30% after the injury. These psychological stress-related symptoms are associated with worse prognoses in SCIs and have been attributed to psychosocial stressors and losses of independence. Nevertheless, emotional and mental modifications after SCI could be related to changes in the volume of specific brain areas associated with information processing and emotions. Additionally, physiological modifications have been recognized as a predisposing factor for mental health depletion, including the development of gut dysbiosis. This condition of imbalance in microbiota composition has been shown to be associated with depression in clinical and pre-clinical models. Therefore, the understanding of the mechanisms underlying the relationship between SCIs, gut dysbiosis and psychological stress could contribute to the development of novel therapeutic strategies to improve SCI patients’ quality of life.

## 1. Introduction

During the last few years, the gut microbiota–brain axis has emerged as a key factor in maintaining homeostasis and influencing central nervous system (CNS) signaling through bidirectional communication. This crosstalk pathway includes afferent and efferent innervation from the autonomic nervous system, the enteric nervous system (an intrinsic neuronal network in the gut mediated by the vagus nerve and sacral innervation) and the hypothalamus pituitary adrenal axis using neural, metabolic, immune and endocrine signaling [1,2,3]. This complex communication contributes to gastrointestinal functions and supports inflammatory responses, behavioral processes, and physiological processes in the CNS including mood, motivation, emotional functions, cognitive functions, brain function (e.g., during brain development, aging, homeostasis, and pathological conditions), protection against pathogens, inflammasome activation, nutrient digestion and absorption, and neurotransmitter production [4,5,6,7]. Brain–gut communication is also regulated by gut microbiota through the modulation of cytokine, neurotransmitter and metabolite production [1,5,8]. Thus, changes in microbiota composition can lead to disturbances in the host–microbiome mutualist relationship, affecting its influence over brain–gut communication and triggering several modifications (e.g., increased inflammatory response and impaired metabolite, vitamin B, and folate and serotonin biosynthesis [7,9,10]). Gut microbiota composition can be shaped by different environmental factors throughout life (e.g., birth method, lifestyle, nutrition, and drug intake) that can contribute to maintaining the homeostasis between the brain and the gut microbiota [2,7]. Critical illness and traumatic conditions have been recognized as modifying factors of gut microbiota composition, causing impairments in intestinal transit, the expression of nutrient transporters, nutritional scarcity, the stability of commensal bacteria (e.g., changes in diversity and interactions), the disruption of mucosal barrier integrity, and alterations of the response to stress mediators [5,11]. Specifically, spinal cord injuries (SCIs) may result in the loss of regulation and control of brain functions, such as for limbs and visceral organs below the level of injury. This has been associated with autonomic dysfunction due to the loss of connections between higher centers and the spinal cord [12,13], including impaired gastrointestinal (GI) tone, gut hormone secretion, a loss of sphincter control, and decreased peristalsis. This consequently leads to intestinal impairment such as SCI-related comorbidities that are able to affect patients’ quality of life [12,14,15] and cause a gut dysbiosis state (i.e., alterations of gut microbiota composition) [16,17,18]. Altered gut microbiota are involved in disease progression and have been indicated as a modifying factor of SCI-related comorbidities [2]. For example, gut dysbiosis has been reported in both humans and animal models of psychological stress [19,20,21], where it has been associated with progression and symptomatology. Psychological morbidities have been reported as one detrimental factor for SCI recovery [22]. Therefore, a better understanding of the crosstalk between the gut microbiota–brain axis and psychological stress following an SCI is required.

In this study, we reviewed selected findings in order to understand the mechanisms underlying their relationship that could contribute to the development of novel therapeutic strategies to improve SCI patients’ quality of life.

## 2. Gut Microbiota

In normal conditions (i.e., homeostasis), the gut microbiome is composed of about 10^14^ microorganisms, which exhibit a great biodiversity. These include bacteria, archaea, virus and eukaryotic species, with a high variability among different individuals but a relatively stable community [10,11,23,24]. Of bacteria, the main phyla are *Firmicutes* (most of them members of the *Clostridia* class, including species related to butyrate production) and *Bacteroidetes* (highlighting 65 characteristic phylotypes). They are followed by *Protobacteria*, *Actinobacteria*, *Fusobacteria* and *Verrucomicrobia* of lesser abundance [25,26,27], with over 160 different bacterial species [28]. Gut microbiota are able to coordinate multiple functions through interactions with other organs to maintain homeostasis (e.g., digestion, nutrient absorption, metabolism, cell development and function within and outside the gastrointestinal tract, and contributions to host immunity) [5,16,23,29,30]. 

The gut microbiota release metabolites produced through fermentation that can act on gut epithelial cells, primary efferent neurons of the GI tract, and the sensitive stimulation of immune cells present in the gut-associated lymphoid tissue (GALT). The latter are able to diffuse into the bloodstream to reach hepatic cells, peripheral immune organs and the CNS [16,23]. Furthermore, gut microbiota bacteria can exert key immunoregulatory functions in order to maintain intestinal homeostasis, such as immune cell priming. This is mediated by stimulating IL-10 production in regulatory T cells (Tregs) in the intestinal epithelium, decreasing inflammation through Tregs activation [31] and therefore limiting pathogens’ exposure in the GI epithelium via the secretion of immunoglobulin A, the promotion of infections defense mediated by the T helper 17 cell (Th17) production of IL-17, and the activation of Th17 [23,30,31]. Furthermore, fermentation-derived short chain fatty acids (SCFAs) such as acetate, propionate, and butyrate are known to be able to affect different cellular functions, e.g., the inhibition of both intestinal stem and progenitor cell proliferation, gut barrier modulation, and inflammation control [23,31]. Furthermore, gut microbiota contribute to tryptophan synthesis and metabolism, regulating its bioavailability for neurotransmitter-producing pathways (such as serotonin and tryptamine), and tryptophan-derived metabolites used for immune response, gut homeostasis and barrier integrity [9,32]. Of note, microbiota function and metabolite production change over lifetime in response to several factors; these variations and interactions with the CNS through gut microbiota–brain axis are further reviewed in [2].

### Gastrointestinal Alterations Following SCI

The term SCI refers to a variety of alterations capable of mainly producing damage to the spinal cord and components of the spinal column, accompanied by immune and autonomic dysfunction, as well as, metabolic and gastrointestinal alterations [16,33,34,35]. SCI causes strong impacts on the physical, psychosocial, and occupational levels [36]. After an SCI, a high rate cell death is triggered along with the disruption of the blood–brain barrier (BBB). This is followed by the infiltration and activation of immune cells, which may lead to the development of partial or total functional alterations (e.g., loss of sensitivity and/or mobility under the level of injury, urinary incontinence, and alteration of both gait and stability) [33,37]. The primary insult of the spinal cord initiates a cascade of events that will lead to the activation of acute and/or chronic mechanisms of injury [37,38]. Injury to the spinal cord can be grouped as trauma-induced and nontraumatic SCIs.

After an SCI, there is a loss of sympathetic preganglionic connections that affects the coordination of spinal cord autonomic function, a situation that could persist for years after an SCI [16]. This autonomic dysfunction may cause impairments of the regulation of autonomic reflexes below the injury level, including those related to the gastrointestinal tract that could causing deficits in colon motility, mucus secretion, vascular tone, and gastric dilatation, as well as decreased gastrointestinal transit [16,29,39]. Gastrointestinal comorbidities have been reported in SCI patients, with a wide variation between general health status and neurological measurements [40]. In SCI patients, 16 s rRNA sequencing from fecal samples showed a reduction in butyrate-producing bacteria compared to healthy controls [41]. Since the innervation of sympathetic preganglionic neurons is distributed between the T5 and T10 segments of the spinal cord, the level of injury may differentially affect their function. This has been reported in both humans and animal models of traumatic SCI [10,42]. For example, using an experimental animal model of SCI, it was shown that alterations in the bacterial and viral communities were more pronounced when SCIs occurred at high levels (T4) compared to low levels (T10) [10]. Furthermore, changes in gastrointestinal structure were reported within three hours after traumatic brain injury in an experimental animal model; specifically, there was epithelial cell tight junction integrity loss [43]. Similarly, a high-thoracic murine SCI model was shown to resemble some of the gastrointestinal clinical features of neurotrauma patients (e.g., reduced gastric emptying and dysmotility) [44,45]. This impairment is partially mediated by alterations in the vagal gastrointestinal response [39]. Altogether, vascular changes, mucus alterations, and structural gastrointestinal tract changes (especially those related to inflammation) will lead to gut microbiota impairment [11]; an imbalance in bacterial gut microbiota composition, such that there is an increase in pathobionts (potentially pathogen bacteria) and a decrease in probionts (beneficial bacteria); and gut dysbiosis [10,29]. This imbalance contributes to the development of systemic inflammatory responses [5] and gut barrier permeabilization, thus producing a bacterial translocation from the intestinal lumen to outside the gastrointestinal tract [29,46]. In addition, modifications in bacterial communities have been shown to cause functional impairments in gut microbiota, with changes in short chain fatty acids (SCFAs) and choline production, including impairments in folate, vitamin B6 and amino acid biosynthesis [10,47,48]. In particular, folate has been shown to play a fundamental role in gastrointestinal and neurologic health, as it has been shown to be related to CNS repairment and regeneration and to contribute to functional recovery and lower neuropathic pain after SCI in animal models [49,50,51]. Furthermore, vitamin B6 dysregulation could produce impairments in the synthesis of neurotransmitters (e.g., serotonin), and the dysfunctional synthesis of the amino acid tryptophan could affect serotonin production, therefore contributing to the development of mental health disorders such as depression, anxiety and fatigue [7,9,10]. As far as we know, there is still a gap knowledge regarding the role of gut microbiota in nontraumatic SCIs. Therefore, this review is focused on traumatic SCIs.

## 3. Psychological Stress in SCI

### 3.1. Demographic Characteristics

A detrimental factor for SCI recovery is an increasing risk of suffering psychological morbidity. It has been estimated that 280 million people worldwide are affected by depression [52]. This number has been expected to raise due to the COVID-19 pandemic. Patients diagnosed with SCIs have a 20–40% risk to develop depression or bipolar disorder during the first months of injury compared to patients without psychiatric comorbidities [22]. Furthermore, depression and anxiety disorders have shown higher prevalences among SCI patients than any other morbidity [53]. The prevalence of depression associated with SCI has shown a wide variation among studies, ranging between 11.9 and 30% [54,55,56,57,58]. Depression has shown an incidence rate ratio of 1.63 in males and 1.19 in females, with higher incidences among people with SCIs between 18 and 35 years old [53]. During the first five years after SCI, a number of risk factors to develop depression disorders have been proposed (e.g., declining health status, increasing pain, low educational level, unemployment, increases in unsafe alcohol consumption, smoking use, antidepressant or stress medication consumption, and the cause of injury) [59,60]. Additionally, lower education levels and unemployment have been related to major depression disorders [56], whereas employability and longer breaks after injury onset have been associated with lower levels of major depression disorders [60]. Ethnicity has not been shown to be related to depression disorder prevalence after SCI, since depressive symptomatology can vary among different population groups [55,60]. Furthermore, the prevalence of anxiety disorders among SCI patients has also shown a wide range of between 2.5 and 31% [61,62]. Self-report measurements have shown a prevalence of up to 27%, and anxiety self-surveys have shown a prevalence range between 15 and 32% after injury [62]. A retrospective analysis conducted in India showed a 40% prevalence of anxiety and a 33% prevalence of depression in studied patients [63]. This suggests the need to develop more studies in this area that can evaluate the effect of depression and antidepressant drugs following SCI.

### 3.2. Causes of Psychological Stress Associated with Spinal Cord Injury

There are a number of risk factors attributed to the development of depression and anxiety disorders after SCI, such as treatment access barriers, late diagnosis, lower educational levels, unemployment, decreased social participation, loss of independence, high health care costs, lifestyle changes, previous mental health history and overweight or obesity, lower quality of life, bladder and bowel dysfunction, caregiver dependency, and low motor score [16,54,56,57,61]. It has also been suggested that the development of psychological morbidities associated with SCI could also follow an independent pathway [59], so the physical changes caused by the injury itself or associated consequences that can direct or indirectly affect mental and emotional status after SCI should be considered during treatment [16].

Furthermore, the reorganization of brain networks and their connectivity has also been described after SCI, whose magnitude of disturbance has been shown to correlate with the initial injury degree. The Salience Network—a brain network related to complex functions such as communication, social behavior, and the integration of sensory, emotional, and cognitive information—has been shown to present the greatest impairments [64,65]. Moreover, functional activity and pattern activation impairments have been reported in the subgenual cingulate, posterior cingulate cortex, and periaqueductal gray matter areas. All of these are brain areas related to emotional processing (e.g., subjective feelings representation and motivational behavior control) [66].

### 3.3. The Gut Microbiota–Brain Axis in Psychological Stress Following SCI

A number of studies have suggested that psychological stresses, such as depression, are accompanied by alterations in gut microbiota composition. For example, depressed patients have shown microbiota alterations, such as decreased richness, phylogenetic diversity, and β-diversity index (related to species diversity between two communities, samples or ecosystems) compared to healthy controls [67]. Furthermore, Jiang et al. reported an increase in α-diversity index (related to species diversity within a community or sample at a small scale) in patients with active major depression disorders compared to healthy controls. For example, *Bacteroidetes* and *Proteobacteria* were found to be more abundant, whereas *Firmicutes* was found to be reduced in patients with active major depression disorders [68]. Pharmacological treatment (e.g., antidepressant drugs) can also alter microbiota composition. Differences were observed at the phylum, family and genus levels between the healthy control group and patients with major depression disorders responsive to pharmacological treatment, without significant differences in richness. Moreover, microbiota composition (abundance of the *Faecalibacterium* genus) was found to negatively correlate with the severity of depressive symptoms [68]. Decreases in richness, phylogenetic diversity, and α-diversity index after fecal microbiota transplant (FMT) from individuals with major depression disorders were observed in a rodent model of antibiotic-induced gut microbiota suppression. These alterations were associated with behavioral changes, such as anxiety-like symptoms and anhedonia [67]. This suggests a bidirectional regulatory effect of the gut microbiota–brain axis on depression. Another study showed that the relative abundances of *Lactobacillus*, *Clostridium* cluster III, and *Anaerofustis* were increased in learned helplessness susceptible rats, accompanied by reduced levels of acetic and propionic acid in the feces, compared to a control group [69]. Furthermore, it has been suggested that the oral administration of *Bifidobacterium* confers resilience to chronic social defeat stress in mice [70], and treatment with a combination of SCFAs was shown to alleviate stress-induced depressive-like symptoms in mice [71]. A study by Hoban et al. showed great depressive-like behavior in rats chronically treated with antibiotic-induced dysbiosis. The specifically observed a decrease in swimming and an increase in immobility scores in the forced swim test. Furthermore, chronically-depleted microbiota showed changes in microbiome diversity following antibiotic treatment—specifically, decreases in *Firmicutes* and *Bacteroidetes* phyla and increases in *Proteobacteria* and *Cyanobacteria* phyla. This condition was also associated with lower serotonin levels, increased serotonin turnover, and altered levels in the dopamine precursor (L-DOPA) in the hippocampus; increased noradrenaline levels in the striatum; and increased tryptophan plasmatic levels [72]. Additionally, impaired neurogenesis in the colonic myenteric has been observed following antibiotic treatment (ampicillin), thus suggesting that antibiotics can also alter the structure and function of the enteric nervous system (e.g., impaired peristalsis) [73]. Schmidt et al. showed the development of anxiety-like symptoms after three weeks of a traumatic incomplete injury in a rat cervical SCI model. Of note, these symptoms were reversed by a gut FMT from healthy rats. Specifically, there was decrease in anxiety-like behavior in the elevated plus maze and light–dark box tests after FMT [74]. Additionally, differences in gut microbiota composition were also reversed following FMT, reaching a normalization between the groups at four weeks after the injury. They additionally observed a correlation between gut microbiota composition and behavioral changes with anxiety-like symptoms [74], thus indicating that alterations in gut microbiota composition could affect the progression of anxiety-like symptoms. Furthermore, probiotics has also been suggested to contribute to the stress resilience response by reducing corticosterone release, anxiety symptoms, and depression symptoms in animal models [75,76], as well as by improving mood disturbances and reducing anxiety symptoms in human patients [77,78,79]. For example, a 12-weeks randomized double-blind and placebo study using the oral administration of *Lactobacillus plantarum* P8 showed a reduction in IFN-γ and TNF-α levels, accompanied by enhanced memory and cognitive functions in treated patients compared to a placebo group [80]. Of note, the cortisol levels did not significantly vary between the two groups. The authors suggested that probiotic treatment attenuates the stress response by diminishing systemic inflammation. Along the same line, pre-treatment with the probiotic mixture of eight strains of OttaBac^®^ (*B. animalis* subsp. *lactis* BL03, BI04; *S. thermophilus* BT01; *Lpb. plantarum* BP06; *L. acidophilus* BA05; *L. helveticus* BD08; *Lcb. paracasei* BP07, and *B. breve* BB02) in a mouse model of systemic inflammation has shown anti-inflammatory properties, specifically by reducing microglia recruitment and activation in the brain, as well as by producing inflammatory cytokines in both the brain and colon [81]. Additionally, other signaling pathways have been suggested to be involved in probiotic modulation. For example, pre-treatment with the *Lactobacillus casei* strain Shirota has been shown to improve mood disturbance in a rat model of water-avoidance stress, reducing corticosterone levels and CRF^+^cFos^+^ double-positive cells in the paraventricular nucleus of the hypothalamus. The authors suggested that *Lactobacillus casei* may be suppressing the hypothalamic–pituitary–adrenal (HPA) axis [82].

### 3.4. Contribution of the Immune Response

Another potential pathway involved in the crosstalk in the gut microbiota–brain axis is the modulation of the immune response by the gut microbiota–brain axis. SCI rats with FMT from anxious donors were shown to exhibit increased anxiety-like behavior compared to the SCI group in an elevated plus maze test. The plasmatic levels of CXCL5 (a chemokine induced by lipopolysaccharides (LPSs) from Gram-negative bacteria) and CCL5 (a chemotactic factor for immune cells), as well as intestinal permeability, were increased in the FMT group compared to a control group [83]. Additionally, anhedonic behavior was also increased in the SCI FMT group compared to the SCI group treated with vehicle solution [83]. Furthermore, SCI rats receiving minocycline treatment showed an attenuated anxious symptomatology compared to an SCI control group in elevated plus maze and light–dark box tests [84]. These results could be partially explained by the anti-inflammatory properties of minocycline at both the systemic and injury levels. Another contributing factor is the direct effect of minocycline treatment on gut microbiota composition after SCI. Specifically, minocycline treatment was found to lead to a decrease in the α-diversity index, transiently affecting the *Firmicutes*/*Bacteroidetes* ratio and therefore modifying gut microbiota function and suggesting a relationship between its anti-inflammatory properties and its anxiolytic effect [84]. Anxiety-like behavior has also been reported in models of thoracic SCI. Wu et al. reported increased anxiety-like behavior in severe and moderate injury groups tested in the open field, as well as depressive-like behavior in sucrose preference and forced swim tests after SCI. These changes were related to severity-dependent neuronal endoplasmic reticule stress in the cortex, hippocampus and thalamic regions, with a lower neuronal density in the thalamus and hippocampus (CA1 and CA2/3 regions) after SCI. The disrupted neurogenesis was also associated with an increase in activated microglia in the cortex and hippocampal regions, as well as increased CCL-21 levels (a neuroimmune modulator chemokine for microglia activation). Furthermore, increased levels of CCL-2 and CCL-3 in the thalamic, hippocampal (C3 and dentate gyrus), and periaqueductal gray regions have been associated with emotional-affective pain responses following SCI through the endocannabinoid pathway [85]. This suggests that neuroinflammation and the activation of the immune system may contribute to psychological stress after SCI, as well as shape gut microbiota composition. Furthermore, impairment in learning and spatial memory have also been found following SCI [86]. Furthermore, Maldonado-Bouchard et al. reported increased serum and spinal cord levels of inflammatory cytokines in both depressed–anxious and depressed-only mice after traumatic thoracic SCI. Specifically, the depressed SCI group exhibited higher levels of TNF-α, CXCL5, GM-CSF, and IL-1β compared to both the depressed–anxious SCI group and the healthy-SCI group. The authors attributed these results to the cytokine hypothesis for the development of depression, which implies that inflammatory cytokines such as TNF-α and IL-6 act in a direct way through (1) tryptophan conversion to kynurenine, decreasing the tryptophan availability for serotonin production; (2) in serotonin degrading from 5-hydroxytryptophan to hydroxyndoleacetic acid, as well as indirectly via cytokines such as TNF-α, IL-1, IL-6, and IFN-γ; and (3) via the activation of the hypothalamus–pituitary–adrenal axis, altering glucocorticoid levels and contributing to hippocampal region inflammation [87]. Similarly, a long-term inflammatory response was reported in female rats, with increased plasmatic levels of TNF-α, IL-1β, IL-6, and IFN-γ and reduced plasmatic levels and spinal cord concentrations of the anti-inflammatory cytokine IL-10, which reverted 28 days after the injury. Furthermore, IL-1β levels were permanently increased but TNF-α, IL-6 and IFN-γ were transiently increased after traumatic thoracic SCI. These changes in the inflammatory response were found to be positively correlated to depression and anhedonia, thus indicating that psychological stress is related to an imbalance between the production and release of inflammatory and anti-inflammatory cytokines, and its persistence over time could contribute to the development of psychiatric disorders after SCI [88]. Rong et al. reported a relationship between gut dysbiosis and the activation of the inflammatory pathway mediated by Toll-like receptor 4 (TLR4). This signaling pathway is activated in response to LPS from Gram-negative bacteria, and it is involved in the destruction of pathogenic bacteria through the release of inflammatory mediators after SCI in mice. The relationship between SCI-induced gut dysbiosis and decreased SCFA-producing phyla has been associated with a greater neuroinflammation. There is a decrease in anti-inflammatory effects on macrophages, where reductions in SCFAs, especially butyrate, could contribute to microglia/macrophage-mediated neurotoxicity [41,42,89]. These results are also consistent with the above-mentioned cytokine hypothesis for the development of depression. 

## 4. Perspective on Future Research

**Figure 1:**
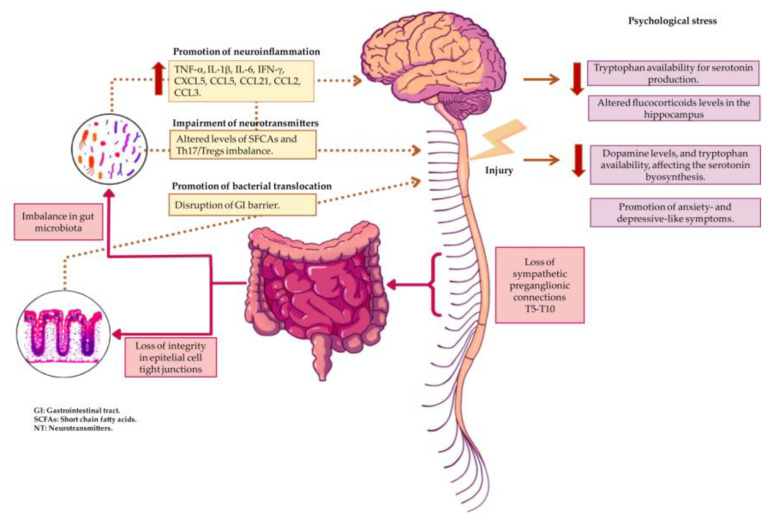
Loss of sympathetic preganglionic connections in thoracic segments T5-T10 after SCI affects the gastrointestinal tract structure. A loss of integrity in epithelial cell tight junctions enables bacterial translocation from gut microbiota, causing inflammation. Furthermore, gut microbiota composition may suffer from an imbalance between pathobionts and probionts bacteria, producing a gut dysbiosis state. This change may contribute to functional impairments in gut microbiota, modifying SCFA production and folate, amino acid, and vitamin B biosynthesis, thus affecting the synthesis of serotonin and increasing neuroinflammation, as well as contributing to the development of mental health disorders. High levels of cytokines and chemokines, as well as disruptions of GI barrier, contribute to the promotion of neuroinflammation. The arrows pointing down indicate decrease, whereas the arrows pointing up indicate increase. NT: neurotransmitters; SCFAs: short-chain fatty acids; aa: amino acids; GI: gastrointestinal; CNS: central nervous system.

An SCI is able to produce multiple impairments, including alterations in gastrointestinal function, leading to gut microbiota imbalance. Gut dysbiosis has been associated with psychological disorders, including bipolar disorder, anxiety, and depression—all of them detrimental factors for SCI prognosis [20,90,91]. Recently, the relationship between gut dysbiosis and psychological stress conditions after SCI has begun to be elucidated through animal models (Figure 1) [74,83,84]. Therefore, it is becoming necessary to further understand its impact on mental health patients’ quality of life. Shorter periods of rest time following injury have been related to increased anxiety and depression symptoms in patients, while longer periods of rest time after the injury have been shown to correlate with lower scores in depressive symptomatology [60,92,93]. Nevertheless, decreases in anxiety and depressive mood over time could be reverted in patients with cognitive impairment following traumatic SCI back to injury stages of acute, discharge, and six months after [94]. This suggests a temporal influence between injury stages and psychological stress impact that should be further researched while considering changes in the gut microbiota changes over time as a possible modifier factor. Nevertheless, future studies require a deep understanding of changes influenced by gut microbiota following SCI, and it will be necessary to not only measure parameter modifications (e.g., diversity, richness, and species characterization) but also to analyze functional gene expression. This will aid the recognition of possible functional changes associated with intestinal dysbiosis, as well as the signaling pathways involved in these processes, hence leading to the understanding of the systemic influence that gut microbiota may exert on the development of psychological stress conditions. For example, probiotic treatments, dietary modifications and appropriate pharmacological management for a balanced gut microbiota composition could be the bases for novel treatment perspectives in SCI patients. Along this line, an experimental animal model probiotic treatment with *Lactobacillus helveticus* and *Bifidobacterium longum* was reported to improve mood and reduce psychological stress. This treatment triggered a rise in tryptophan levels and a decrease in inflammation [95]. Similarly, in a pilot study performed in patients with irritable bowel syndrome, treatment with *Bifidobacterium longum* was shown to reduce depression-like symptoms and fearful stimuli in brain areas related to processing emotions, as well as to improve the physical domain of quality of life [96]. Furthermore, in a mouse model of SCI, the administration of probiotics enriched with lactic-producing bacteria promoted the recovery of locomotor function and improved immune function [46]. Recently, a single large-scale homogeneous population-based cohort of 6000 Finnish people (FINRISK study) suggested a possible link between depression and the abundance of *Morganella* and *Kiebdsiella (*ex*-Raoltella*) [97]. The researchers combined the use of matching human genotypes and shotgun fecal metagenomic results to perform a causal interference analysis [98]. Hence, further research aiming to elucidate the complexity in the interactions in the microbiota–brain–gut axis in SCI will be crucial for the development of novel therapeutic strategies with greater specificity, aiming to restore microbiota balance with the potential to restrict the development of psychological stress conditions that are detrimental for SCI progression and recovery. SCI-related factors, such as injury level, completeness, and severity, could play major roles in gut microbiota composition [10,41,89] and therefore produce negative impacts on psychological stress. In this regard, a recent study using an animal model of traumatic SCI showed that high versus low injury levels trigger differences in microbiota composition (T4 versus T10) [10]. Thus, to obtain a deeper comprehension of the relationships between SCI, gut microbiota, and psychological stress conditions, studies that include psychological stress assessments while considering SCI-related factors could provide valuable information to elucidate the potential feedback loop in the brain–gut axis. Future treatment options should also account for the effect of gut microbiota in the availability of pharmacological drugs or how antidepressant drugs can shape gut microbiota composition, as well as the effect of gut microbiota on the production of neurotransmitters such as serotonin, the levels of which are reduced after depression [99]. It is known that antidepressant drugs have antimicrobial properties [100]. However, their mechanism of action is not completely understood in some cases. For example, the authors of an in vitro study showed that desipramine (a tricyclic antidepressant) can alter gut microbiota composition with the most potent antibacterial activity [101]. Furthermore, lithium, valproate, and aripiprazole have been shown to increase microbial richness and diversity, whereas escitalopram, venlafaxine, fluoxetine, and aripraxole can increase gut permeability [102]. One of the current limitations of our understanding of how antidepressant drugs can modulate gut microbiota is based the lack of in vivo studies assessing the effect of antidepressant drugs on human gut microbiota. Most of the current information comes from studies performed in animal models.

## Data Availability

Not applicable.
